# Slx8 Removes Pli1-Dependent Protein-SUMO Conjugates Including SUMOylated Topoisomerase I to Promote Genome Stability

**DOI:** 10.1371/journal.pone.0071960

**Published:** 2013-08-06

**Authors:** Roland Steinacher, Fekret Osman, Alexander Lorenz, Claire Bryer, Matthew C. Whitby

**Affiliations:** Department of Biochemistry, University of Oxford, Oxford, United Kingdom; National Cancer Institute, United States of America

## Abstract

The SUMO-dependent ubiquitin ligase Slx8 plays key roles in promoting genome stability, including the processing of trapped Topoisomerase I (Top1) cleavage complexes and removal of toxic SUMO conjugates. We show that it is the latter function that constitutes Slx8's primary role in fission yeast. The SUMO conjugates in question are formed by the SUMO ligase Pli1, which is necessary for limiting spontaneous homologous recombination when Top1 is present. Surprisingly there is no requirement for Pli1 to limit recombination in the vicinity of a replication fork blocked at the programmed barrier *RTS1*. Notably, once committed to Pli1-mediated SUMOylation Slx8 becomes essential for genotoxin resistance, limiting both spontaneous and *RTS1* induced recombination, and promoting normal chromosome segregation. We show that Slx8 removes Pli1-dependent Top1-SUMO conjugates and in doing so helps to constrain recombination at *RTS1*. Overall our data highlight how SUMOylation and SUMO-dependent ubiquitylation by the Pli1-Slx8 axis contribute in different ways to maintain genome stability.

## Introduction

The posttranslational modification of proteins with ubiquitin and Small Ubiquitin-like MOdifier (SUMO) plays an important role in promoting and coordinating DNA repair [1], [2], [3]. SUMO conjugation to proteins can modulate their DNA binding, enzymatic turnover, interaction with other proteins, subcellular localization and stability [4], [5], [6], [7], [8], [9]. SUMO is covalently attached to conserved lysine residues of target proteins by an enzymatic cascade, which involves an activating enzyme (E1), a conjugating enzyme (E2) and a protein ligase (E3) [10]. In the fission yeast *Schizosaccharomyces pombe* two SUMO E3 protein ligases, Pli1 and Nse2, have been identified [11], [12]. Pli1 is the major SUMO ligase being responsible for most of the SUMO conjugates detected in cell extracts [13]. It is important for telomere maintenance, but not for the repair of genotoxin-induced DNA damage [12], [13]. Nse2 is part of the Smc5-Smc6 complex and promotes DNA repair [14].

The discovery of the SUMO-targeted ubiquitin ligase (STUbL) Slx8 revealed that there is interplay between the SUMO and ubiquitylation pathways. Slx8 was shown to ubiquitylate SUMOylated proteins to mark them for proteasomal degradation [13], [15], [16], [17], [18], [19], [20], [21]. This function appears to play an important role in ensuring genome stability during DNA replication, since Slx8 colocalizes with PCNA in replication foci and limits recombination at the programmed replication fork barrier of the rDNA locus [22]. Recent work in the fission yeast *Schizosaccharomyces pombe* demonstrated that Nse2/Slx8 mediated SUMOylation/Ubiquitylation functions to suppress spontaneous Topoisomerase I (Top1) mediated genome instability [23].

Top1 plays an important role in the relaxation of supercoiled DNA that forms ahead of both the replication and transcription machinery [24]. It does this by cleaving one DNA strand to generate a covalent protein-DNA intermediate, the so-called Top1cc, which can then rotate around the intact complementary strand. Rounds of strand rotation are usually followed by the re-ligation of the single strand nick, however, in the presence of DNA lesions, such as single-strand breaks and abasic sites, or the Top1 poison camptothecin (CPT), re-ligation is inhibited resulting in the persistence of the Top1cc, which in turn can inhibit transcription and lead to replication fork stalling and chromosome breakage [25], [26], [27]. Thus mechanisms for the removal of trapped Top1cc are essential for ensuring genome stability. In *S. pombe* processing of Top1cc appears to rely on either the tyrosyl-DNA phosphodiesterase Tdp1 or a pathway involving Nse2, Slx8 and the SUMO mimetic Rad60, which are thought to somehow promote the activity of the nucleotide excision repair endonuclease Rad16-Swi10 in removing Top1cc [23].

Top1 is SUMOylated by Pli1, however Pli1 is seemingly not required for processing Top1cc and the function of this SUMOylation in fission yeast remains unclear [23]. Intriguingly the presence of Top1 without Pli1 (or in budding yeast Siz1 and Siz2) engenders a dependency on homologous recombination (HR) factors, including Rad51, for cell viability [28], [29]. This suggests that SUMOylation of Top1 and/or other proteins is needed to govern a Top1-dependent process, which otherwise necessitates the need for HR. Interestingly Pli1-dependent SUMOylation also necessitates a requirement for Slx8 to prevent the accumulation of toxic SUMO chains [13], [28].

Here we confirm that the presence of Top1 results in a need for Pli1-dependent SUMOylation to limit spontaneous recombination. Intriguingly, Pli1 SUMOylation is dispensable at a programmed replication fork barrier *RTS1*, which is a potential recombination hotspot [30], [31], [32], [33], [34]. We show that in the absence of Slx8, SUMOylated proteins, including Top1, accumulate in a Pli1-dependent manner. Although the failure to process SUMOylated Top1 by Slx8 is not the only cause of the heightened spontaneous genome instability and reduced cell viability, it significantly contributes to elevated recombination levels when forks stall at the programmed replication barrier *RTS1*.

## Materials and Methods

### *S. pombe* strains

*S. pombe* strains are listed in Table 1. The *slx8* deletion strain was made by PCR-based gene targeting [35].

**Table 1:** *S. pombe* strains used in this study.

Strain	Relevant genotype
MCW1221	*h^+^ ura4-D18 leu1-32 his3-D1 arg3-D4*
FO986	*h^+^ slx8*Δ::*kanMx6 ura4-D18 leu1-32 his3-D1 arg3-D4*
MCW4568	*h^−^ pli1Δ::ura4^+^ ura4-D18 leu1-32 his3-D1 arg3-D4*
MCW4688	*h^+^ pli1Δ::ura4^+^ slx8Δ::kanMx6 ura4-D18 leu1-32 his3-D1 arg3-D4*
MCW5057	*h^+^ nse2-SA::ura4^+^ ura4-D18 leu1-32 his3-D1 arg3-D4*
MCW5122	*h^+^ nse2-SA::ura4^+^ slx8Δ::kanMx6 ura4-D18 leu1-32 his3-D1 arg3-D4*
MCW5663	*h^+^ pli1Δ::kanMx6 nse2-SA::ura4^+^ ura4-D18 leu1-32 his3-D1 arg3-D4*
MCW6514	*h^+^ top1*Δ::*natMx4 slx8*Δ::*kanMx6 ura4-D18 leu1-32 his3-D1 arg3-D4*
MCW6516	*h^+^ top1*Δ::*natMx4 ura4-D18 leu1-32 his3-D1 arg3-D4*
MCW4712	*h^+^ ura4-D18 leu1-32 his3-D1 arg3-D4 ade6-M375 int*::*pUC8/his3^+^/RTS1 site A orientation 1/ade6-L469*
MCW4713	*h^+^ ura4-D18 leu1-32 his3-D1 arg3-D4 ade6-M375 int*::*pUC8/his3^+^/RTS1 site A orientation 2/ade6-L469*
MCW4774	*h^+^ pli1Δ::ura4^+^ ura4-D18 leu1-32 his3-D1 arg3-D4 ade6-M375 int::pUC8/his3^+^/RTS1 site A orientation 1/ade6-L469*
MCW4776	*h^+^ pli1Δ::ura4^+^ ura4-D18 leu1-32 his3-D1 arg3-D4 ade6-M375 int::pUC8/his3^+^/RTS1 site A orientation 2/ade6-L469*
MCW5131	*h^+^ nse2-SA::ura4^+^ ura4-D18 leu1-32 his3-D1 arg3-D4 ade6-M375 int::pUC8/his3^+^/RTS1 site A orientation 1/ade6-L469*
MCW5133	*h^+^ nse2-SA::ura4^+^ ura4-D18 leu1-32 his3-D1 arg3-D4 ade6-M375 int::pUC8/his3^+^/RTS1 site A orientation 2/ade6-L469*
MCW5463	*h^+^ pli1Δ::kanMx6 nse2-SA::ura4^+^ ura4-D18 leu1-32 his3-D1 arg3-D4 ade6-M375 int::pUC8/his3^+^/RTS1 site A orientation 1/ade6-L469*
MCW5466	*h^+^ pli1Δ::kanMx6 nse2-SA::ura4^+^ ura4-D18 leu1-32 his3-D1 arg3-D4 ade6-M375 int::pUC8/his3^+^/RTS1 site A orientation 2/ade6-L469*
MCW4826	*h^+^ slx8Δ::kanMx6 ura4-D18 leu1-32 his3-D1 arg3-D4 ade6-M375 int::pUC8/his3^+^/RTS1 site A orientation 1/ade6-L469*
MCW4828	*h^+^ slx8Δ::kanMx6 ura4-D18 leu1-32 his3-D1 arg3-D4 ade6-M375 int::pUC8/his3^+^/RTS1 site A orientation 2/ade6-L469*
MCW4830	*h^+^ pli1Δ::ura4+ slx8Δ::kanMx6 ura4-D18 leu1-32 his3-D1 arg3-D4 ade6-M375 int::pUC8/his3^+^/RTS1 site A orientation 1/ade6-L469*
MCW4832	*h^+^ pli1Δ::ura4+ slx8Δ::kanMx6 ura4-D18 leu1-32 his3-D1 arg3-D4 ade6-M375 int::pUC8/his3^+^/RTS1 site A orientation 2/ade6-L469*
MCW6560	*h^+^ top1Δ::natMx4 ura4-D18 leu1-32 his3-D1 arg3-D4 ade6-M375 int::pUC8/his3^+^/RTS1 site A orientation 1/ade6-L469*
MCW6093	*h^+^ top1Δ::natMx4 ura4-D18 leu1-32 his3-D1 arg3-D4 ade6-M375 int::pUC8/his3^+^/RTS1 site A orientation 2/ade6-L469*
MCW5631	*h^+^ top1Δ::LEU2^+^ pli1Δ::ura4^+^ ura4-D18 leu1-32 his3-D1 arg3-D4 ade6-M375 int::pUC8/his3^+^/RTS1 site A orientation 1/ade6-L469*
MCW5633	*h^−^ top1Δ::LEU2^+^ pli1Δ::ura4^+^ ura4-D18 leu1-32 his3-D1 arg3-D4 ade6-M375 int::pUC8/his3^+^/RTS1 site A orientation 2/ade6-L469*
MCW6549	*h^+^ top1Δ::natMx4 slx8Δ::kanMx6 ura4-D18 leu1-32 his3-D1 arg3-D4 ade6-M375 int::pUC8/his3^+^/RTS1 site A orientation 1/ade6-L469*
MCW6551	*h^−^ top1Δ::natMx4 slx8Δ::kanMx6 ura4-D18 leu1-32 his3-D1 arg3-D4 ade6-M375 int::pUC8/his3^+^/RTS1 site A orientation 2/ade6-L469*
MCW5987	*h^+^ top1-myc::natMx4 ura4-D18 leu1-32 his3-D1 arg3-D4*
MCW6242	*h^−^ top1-myc::natMx4 pli1Δ::ura4^+^ ura4-D18 leu1-32 his3-D1 arg3-D4*
MCW6345	*h^+^ top1-myc::natMx4 slx8Δ::kanMx6 ura4-D18 leu1-32 his3-D1 arg3-D4*
MCW6284	*h^+^ top1-myc::natMx4 pli1Δ::ura4^+^ slx8Δ::kanMx6 ura4-D18 leu1-32 his3-D1 arg3-D4*

### Media and genetic methods

Media and genetic methods followed standard protocols [36]. The complete and minimal media were yeast extract with supplements (YES) and Edinburgh minimal medium plus 3.7 mg/ml sodium glutamate (EMMG) plus appropriate amino acids (0.25 mg/ml), respectively. Low adenine media (YELA) was supplemented with 0.01 mg/ml adenine. Ade^+^ recombinants were selected on YES lacking adenine and supplemented with 0.2 mg/ml guanine to prevent uptake of residual adenine.

### Spot assays

Exponentially growing cells from liquid cultures were harvested, washed and resuspended in water at a density of 1×10^7^–1×10^3^ cells/ml. Aliquots (10 µl) of the cell suspensions were spotted onto agar plates containing genotoxins as indicated. For UV, plates were irradiated using a Stratalinker (Stratagene). Plates were photographed after 5–7 days growth at 25°C, 30°C or 37°C as indicated.

### Microscopy

Cells from asynchronously growing cultures were fixed with 70% ethanol and stored at 4°C for later analysis. Fixed cells were rehydrated, stained with DAPI and then analysed using an Olympus BX50 epifluorescence microscope equipped with the appropriate filter set to detect blue fluorescence (Chroma Technology Corp., VT). Black and white images were acquired with a CoolSNAP HQ CCD camera (Photometrics, AZ) controlled by MetaMorph software (v7.7.3.0, Molecular Devices Inc., CA).

### Recombination assays

The direct repeat recombination assay was performed as described [30], [37], [38]. Two sample *t* tests were used to determine the statistical significance of differences in recombination values between strains.

### Western blots

Whole-cell protein extracts were made from asynchronously growing yeast cultures as described [39]. Western blots were probed with rabbit anti-Pmt3 (a gift from F. Watts), mouse anti-tubulin (Sigma), and mouse anti-C-myc (Sigma) antibodies as indicated.

### 2D gels

The protocol for analysis of replication intermediates by 2D gel electrophoresis has been described previously [40].

## Results

### Pli1-dependent SUMOylation in the absence of Slx8 results in reduced cell viability, hypersensitivity to genotoxins and defects in chromosome segregation

**Figure 1:**
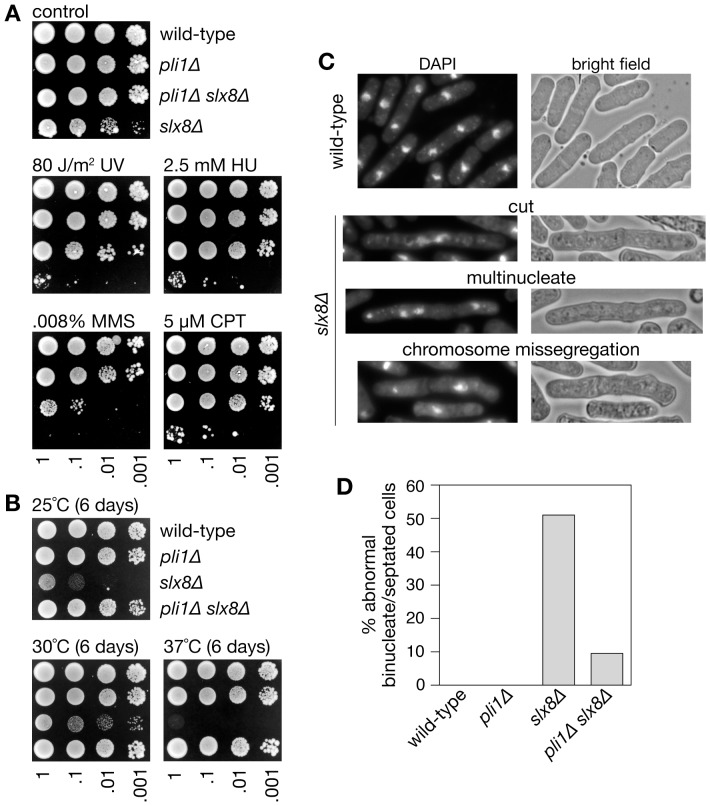
Cell growth, genotoxin sensitivity and chromosome segregation in *slx8* Δ and *pli1*Δ single and double mutants. (**A**) Spot assay comparing the genotoxin sensitivities of strains MCW1221, MCW4568, MCW4688 and FO986. Plates were photographed after 6 days growth at 30°C. (**B**) Spot assay comparing the growth at different temperatures of the same strains as in A. Plates were photographed after 6 days growth at the indicated temperature. (**C**) Example images of wild-type and *slx8*Δ binucleate and septated cells. (**D**) Percentage of binucleate and septated cells exhibiting abnormal chromosome segregation. A total of 100 binucleate/septated cells from two independent cultures were analysed for each strain.

**Figure 2:**
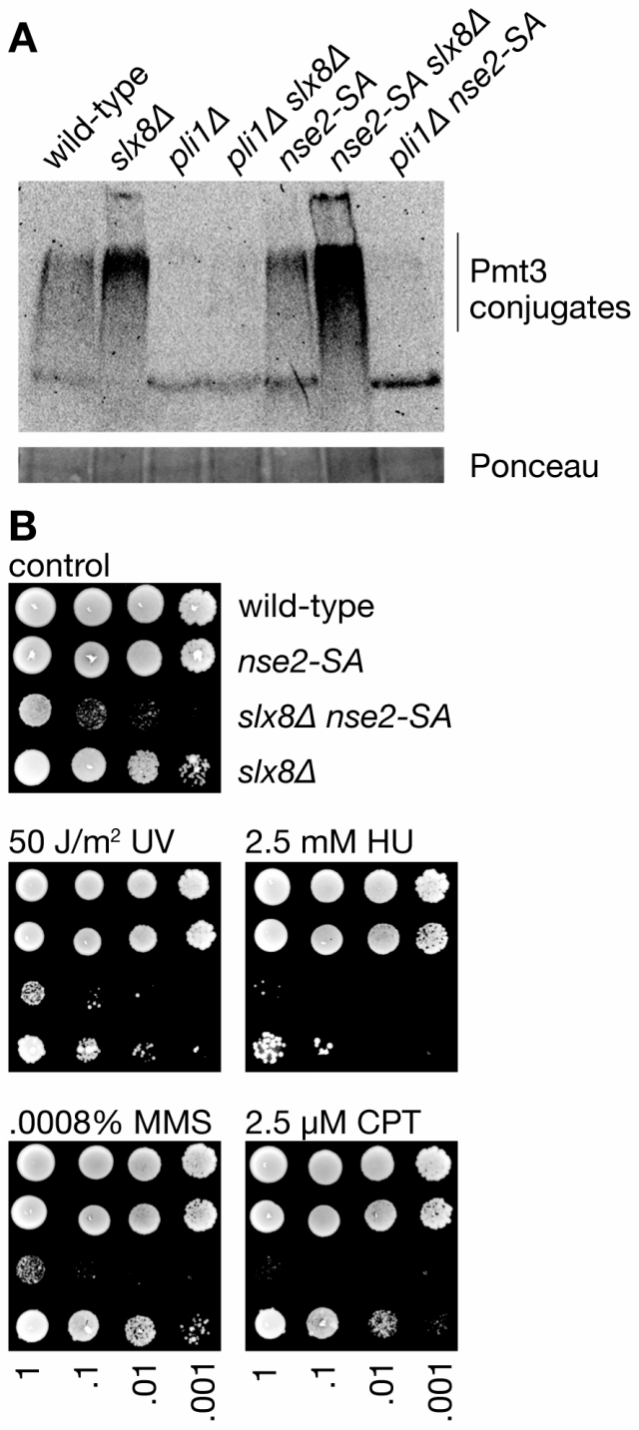
SUMO conjugates accumulate in *slx8* Δ and *slx8*Δ* nse2-SA* mutant cells and correlates with reduced growth and hypersensitivity to genotoxins. (**A**) Western blot of SUMO (Pmt3) conjugates in whole-cell extracts from asynchronously growing cultures of strains MCW1221, FO986, MCW4568, MCW4688, MCW5057, MCW5122 and MCW5663. (**B**) Spot assay comparing the growth and genotoxin sensitivity of strains MCW1221, MCW4568, MCW5122 and FO986. Plates were photographed after 7 days growth at 30°C.

The biological importance of Slx8 in *S. pombe* has mainly been investigated using a hypomorphic temperature-sensitive mutant, and therefore experiments have involved a temperature shift to study a partially impaired Slx8 protein at the restrictive temperature (36°C) [13]. Heat shock can induce SUMOylation and may lead to the formation of specific SUMO conjugates as a stress response [41], [42]. Whether Slx8 preferentially targets such stress-induced SUMO conjugates or SUMO conjugates in general is not entirely clear. To be able to characterize the effects of Slx8 deficiency on SUMOylation at 30°C we constructed a strain in which the *slx8* gene was fully deleted. The *slx8*Δ mutant is viable but exhibits slow growth, elongated cells, temperature sensitivity (at 37°C) and hypersensitivity to ultraviolet light (UV), hydroxyurea (HU), CPT and the alkylating agent methyl methanesulfonate (MMS) (Fig. 1A and B). Strikingly, deletion of *pli1* in the *slx8*Δ background fully or partially suppressed all of these phenotypes (Fig. 1A and B). Analysis of cells stained with the DNA-specific dye DAPI revealed a high percentage of binucleate and septated *slx8*Δ cells with abnormalities, including cut phenotypes, missegregated chromosomes and multinucleated cells (Fig. 1C and D). Again these phenotypes are largely suppressed by deleting *pli1* (Fig. 1D). Western blot analysis of whole-cell extracts from asynchronously growing yeast cultures showed an accumulation of SUMOylated protein conjugates in *slx8*Δ cells compared to wild-type (Fig. 2A). In *pli1*Δ cells SUMOylation was barely detectable, which is consistent with an earlier report [43]. Likewise SUMOylation was virtually absent in the *pli1Δ slx8*Δ double mutant (Fig. 2A). These results suggest that Slx8 is needed to remove Pli1-dependent SUMO conjugates under normal growth conditions, and in its absence these SUMO conjugates accumulate and cause toxicity.

### Loss of fitness in *nse2-SA slx8*Δ mutant cells correlates with aberrant hyper-SUMOylation

Next we investigated whether deficiency in Nse2-mediated SUMOylation also alleviates the poor growth and genotoxin sensitivity of *slx8*Δ cells. As Nse2 is an essential protein we made use of the Nse2-SA mutant, which is deficient in SUMO conjugation [14]. Unlike the *pli1Δ slx8*Δ double mutant, the *nse2-SA slx8*Δ double mutant exhibits a synergistic reduction in growth and increased hypersensitivity to CPT and MMS when compared to its parental single mutant strains (Fig. 2B). Intriguingly when we analysed the level of SUMO conjugates in the double mutant we observed that they accumulated to even higher levels than in a *slx8*Δ single mutant (Fig. 2A). This suggests that in the absence of Nse2, Pli1-dependent SUMOylation is further stimulated and may even start to act on Nse2 target proteins. This aberrant hyper-SUMOylation is probably the cause of the severe growth defect and genotoxin sensitivity of the *nse2-SA slx8*Δ double mutant.

### Pli1 and Slx8 limit spontaneous recombination

**Figure 3:**
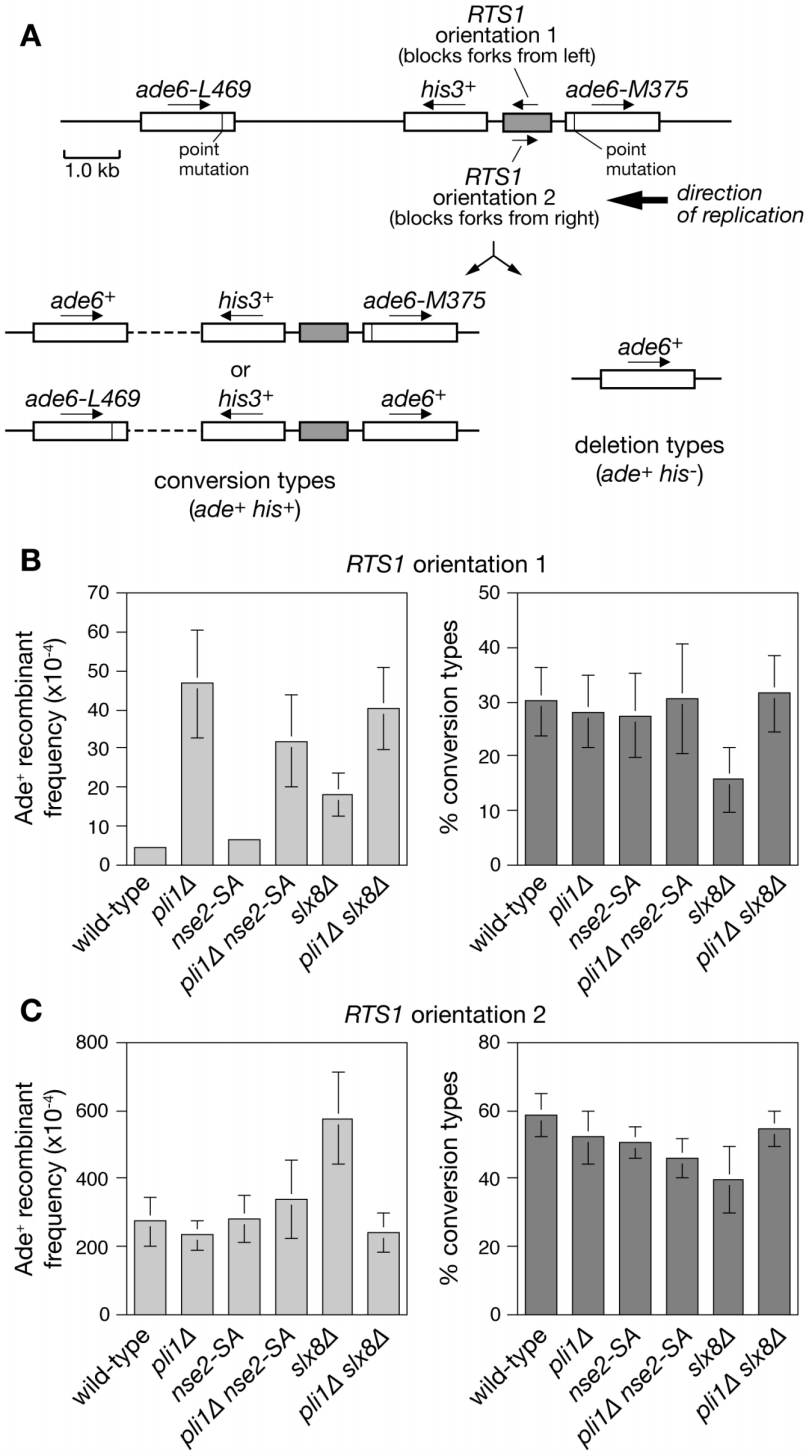
Spontaneous and *RTS1*-induced direct repeat recombination in cells deficient for SUMOylation and/or STUbL activity. (**A**) Schematic of the *ade6^−^* direct repeat on chromosome 3 and two classes of Ade^+^ recombinant. (**B** and **C**) Ade^+^ recombinant frequencies (left panels) and the percentage of recombinants that are conversion types (right panels). The strains are MCW4712, MCW4774, MCW5131, MCW5463, MCW4826, MCW4830, MCW4713, MCW4776, MCW5133, MCW5466, MCW4828 and MCW4832. Error bars are the standard deviations about the mean.

Previous studies have shown that deletion of either *slx8* or *siz1* and *siz2*, which encode SUMO E3 ligases, results in increased levels of spontaneous mitotic recombination in budding yeast [22], [29]. Similarly loss of Pli1 results in hyper-recombination in fission yeast [44]. However, the *slx8* temperature sensitive mutant (*slx8-1*) has only been tested at its permissive temperature where it exhibits no significant change in direct repeat recombination compared to wild-type [23]. To clarify the relative importance of Pli1-dependent SUMOylation and Slx8-dependent SUMO-targeted ubiquitylation for limiting recombination in fission yeast we used strains harbouring a direct repeat of *ade6^−^* heteroalleles with an intervening *his3^+^* gene and *RTS1* element (Fig. 3A) [30], [37]. In these strains the frequency of recombination between the two copies of *ade6* can be monitored by the appearance of Ade^+^ prototrophs, which arise either from a gene conversion event or from a deletion event, with the former being distinguished from the latter by the retention of the *his3^+^* gene. *RTS1* is a strong polar replication fork barrier (RFB), which when positioned in orientation 2 blocks replication forks traversing the *ade6* locus causing a large increase in direct repeat recombination (Fig. 3A) [30], [37]. However, due to the placement of replication origins, the *ade6* locus is replicated unidirectionally and therefore when positioned in orientation 1 *RTS1* does not impede the passage of the replication fork and consequently has no effect on the local frequency of recombination [30], [37], [45]. In wild-type cells with the non-blocking orientation of *RTS1* (i.e. orientation 1), the Ade^+^ prototroph frequency is ∼4 in 10,000 viable cells with a conversion-type to deletion-type ratio of 3∶7 (Fig. 3B). The *nse2-SA* mutant exhibited similar levels of recombination as the wild-type, whereas Pli1 and Slx8 deficient cells showed an approximately 12-fold (*P*<0.01) and 5-fold (*P*<0.01) increase in Ade^+^ frequency, respectively (Fig. 3B and Table S1). The conversion-type to deletion-type ratio in both *nse2-SA* and *pli1*Δ mutants was unchanged, but in *slx8*Δ cells the proportion of deletion-types was increased (conversion-type to deletion-type ratio 1.5∶8.5) (*P*<0.01) (Fig. 3B and Table S1). Conversion-types depend on Rad51 for their formation, whereas deletion-types can arise by Rad51-independent pathways such as single strand annealing (SSA), which is dependent on Rad52 (formerly known as Rad22) [46]. However, we were unable to explore the genetic dependency of the hyper-recombination in a *slx8*Δ mutant as both *slx8Δ rad51*Δ and *slx8Δ rad52*Δ double mutants were not viable (data not shown). The *pli1Δ nse2-SA* and *pli1Δ slx8*Δ double mutants produced a similar Ade^+^ prototroph frequency and conversion-type to deletion-type ratio as the *pli1*Δ single mutant (Fig. 3B and Table S1). These results indicate that Pli1-dependent SUMOylation and Slx8-dependent processing of SUMO conjugates function in a common pathway for limiting spontaneous recombination, with the former process being more important than the latter for restricting the overall number of recombinants that are formed. In contrast Nse2-dependent SUMOylation appears to have relatively little impact on the frequency of direct repeat recombination.

### Slx8 promotes genome stability at the polar replication fork barrier *RTS1*

**Figure 4:**
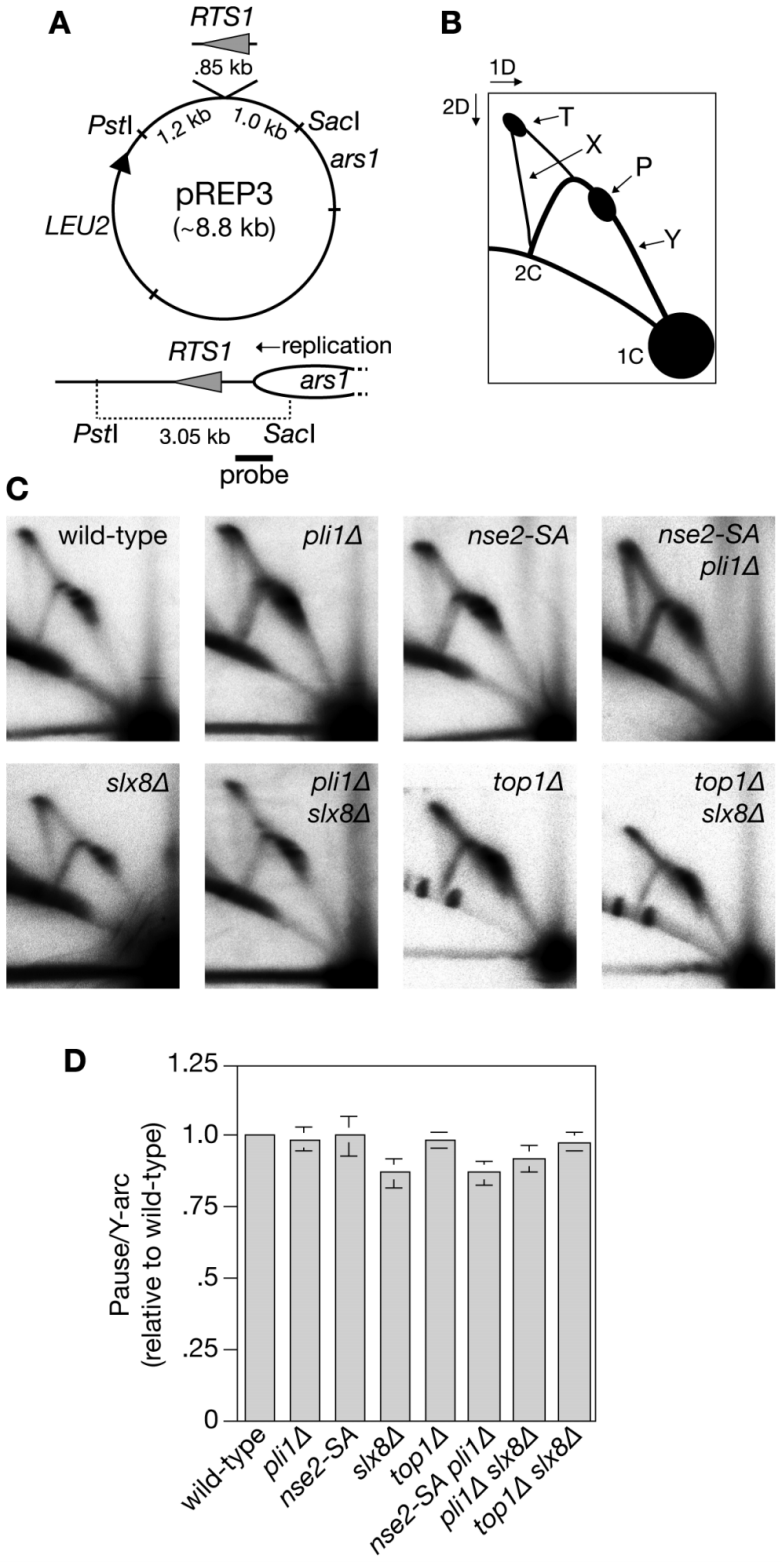
Analysis of replication fork blockage at *RTS1* on a plasmid in cells deficient for SUMOylation, STUbL activity and Top1. (**A**) Schematic of plasmid pREP3 containing *RTS1*, which is orientated so that it blocks the replication fork that approaches it from the replication origin (*ars1*) on its right as drawn [66]. The bottom panel shows a replication fork originating from *ars1* moving toward *RTS1* and the position of the probe used for the analysis in C. (**B**) Schematic showing the main features of the 2D gel analysis of replication intermediates in C. These are: the arc of Y-shaped replication forks (Y); replication forks blocked/paused at *RTS1* (P); replication termination where two opposing forks merge (T); and a spike of X-shaped DNA molecules that represent fully replicated conjoined DNAs. (**C**) 2D gel analysis of replication intermediates in the PstI-SacI fragment shown in A from wild-type or mutant cells as indicated. The strains are MCW1221, MCW4568, MCW5057, FO986, MCW6516, MCW5663, MCW4688 and MCW6514. (**D**) Amount of replication fork blockage/pausing as a percentage of the total Y-arc relative to wild-type. Values are the means of three independent experiments. Error bars represent standard deviations.

Having established that both Pli1 and Slx8 play an important role in limiting spontaneous recombination we next tested whether they are similarly important for restricting recombination that is induced by replication fork blockage at *RTS1*. In line with previous data *RTS1* in orientation 2 causes a ∼65-fold increase in Ade^+^ frequency compared to the spontaneous level of recombination, with slightly more than half of the recombinants being conversion-types [30] (Fig. 3C and Table S1). Similar to what we observed for spontaneous recombination the *nse2-SA* mutant exhibited no significant change in recombinant frequency, whereas the *slx8*Δ mutant showed a ∼2-fold increase (*P*<0.01) with a slight bias towards deletion-type recombinants (*P*<0.01) (Fig. 3C and Table S1). Surprisingly, and in marked contrast to its effect on spontaneous recombination, deletion of *pli1* had little effect on the level of *RTS1*-induced recombinants (Fig. 3C and Table S1). The same is true for a *pli1 nse2-SA* double mutant indicating that the lack of a marked effect is not due to redundancy between the two E3 ligases (Fig. 3C and Table S1). A previous study found a dependency on SUMO for establishment/maintenance of the *RTS1* RFB [47]. However, analysis of replication fork blockage at *RTS1* by native two-dimensional gel electrophoresis showed that neither *pli1*Δ nor *nse2-SA* (either as single or double mutants) caused a significant reduction in barrier strength (Fig. 4A–D). Importantly, the hyper-recombination in a *slx8*Δ mutant is reduced to wild-type levels by deletion of *pli1*, and again this effect is not due to an alteration in barrier strength (Fig. 3C and 4C–D). Together these data show that neither Pli1- nor Nse2-dependent SUMOylation play a major role in promoting or limiting recombination that is induced by replication fork blockage at *RTS1*. However, once cells are committed to Pli1-dependent SUMOylation, Slx8 is needed here to constrain recombination.

### Deletion of Top1 suppresses the hyper-recombination in a *pli1*Δ mutant

**Figure 5:**
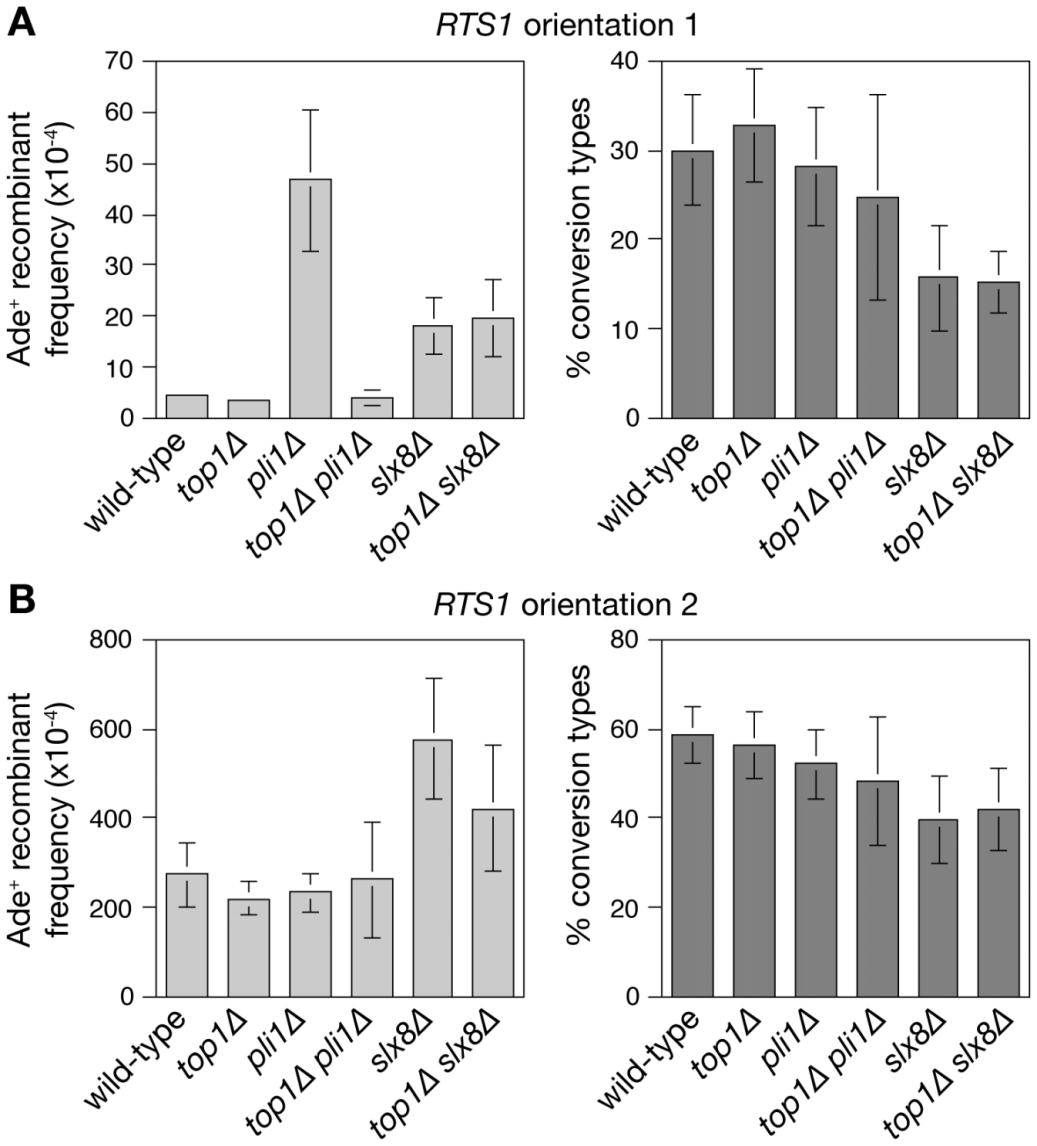
Effect of *top1* deletion on spontaneous and *RTS1*-induced direct repeat recombination in *pli1* Δ and *slx8*Δ mutants. (**A** and **B**) Ade^+^ recombinant frequencies (left panels) and the percentage of recombinants that are conversion types (right panels). The strains are MCW4712, MCW4774, MCW6560, MCW5631, MCW4826, MCW6549, MCW4713, MCW4776, MCW6093, MCW5633, MCW4828 and MCW6551. Error bars are the standard deviations about the mean.

As discussed above, HR factors such as Rad51 are important for cell viability in the absence of Pli1-dependent SUMOylation (or Siz1/Siz2-dependent SUMOylation in budding yeast), which is consistent with the elevated recombination observed in *pli1*Δ and *siz1Δ siz2*Δ mutant cells (Fig. 3B) [29], [44]. Intriguingly the inviability of a *pli1Δ rad51*Δ double mutant and growth defects of a *siz1Δ siz2Δ rad51*Δ triple mutant appear to be caused by Top1 activity, however surprisingly the hyper-recombination of a *siz1Δ siz2*Δ mutant in budding yeast is not suppressed by deleting *top1* [28], [29]. In contrast, the elevated level of spontaneous recombination in a *pli1*Δ mutant is fully suppressed when *top1* is deleted (Fig. 5A and Table S1).

### SUMOylated Top1 accumulates in a *slx8*Δ mutant

**Figure 6:**
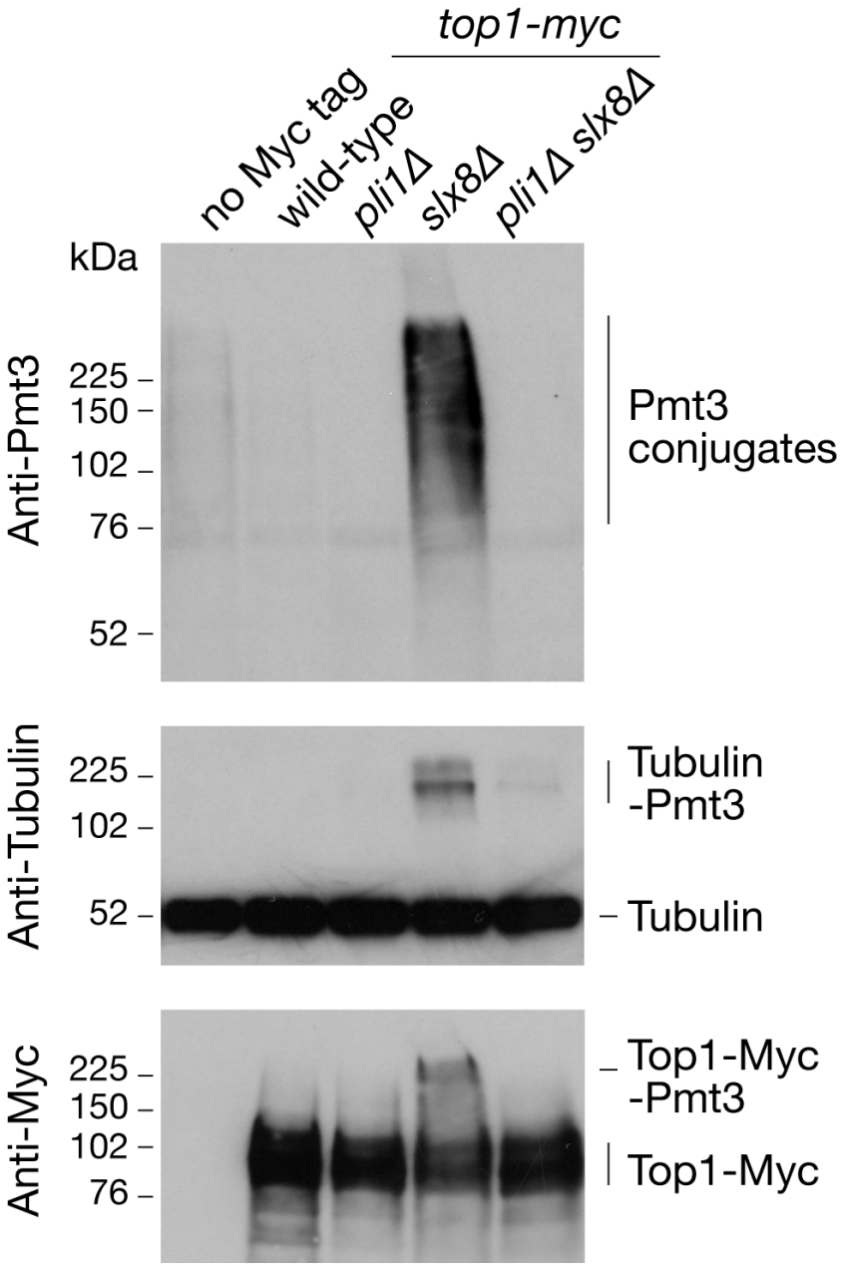
Top1-SUMO accumulates in a *slx8*Δ mutant. Western blots analysing total SUMO conjugates, tubulin and Top1-Myc in whole-cell extracts from asynchronously growing cultures of strains MCW1221, MCW5987, MCW6242, MCW6345 and MCW6284.

The aforementioned data imply that Pli1-dependent SUMOylation prevents Top1 from causing a need for HR. One way it could achieve this is by marking Top1 for degradation through the Slx8-dependent pathway. This idea has been largely discounted as western blot analysis showed that SUMOylated Top1 does not accumulate in a *slx8* temperature sensitive mutant [23]. However, using a strain expressing Myc-tagged Top1 we were able to detect SUMO conjugated Top1 in a *slx8*Δ mutant, but not in wild-type, *pli1*Δ or *pli1Δ slx8*Δ strains (Fig. 6). This suggests that Pli1-dependent SUMOylation does target Top1 for Slx8-dependent degradation. Interestingly we also observed that tubulin, which was used as a loading control and is known to be SUMOylated in budding yeast and human cells [48], [49], likewise accumulates in a high molecular weight SUMOylated form in a *slx8*Δ mutant, and that this is again dependent on Pli1 (Fig. 6).

### Removal of Top1-SUMO conjugates by Slx8 helps to limit local hyperrecombination at *RTS1*

**Figure 7:**
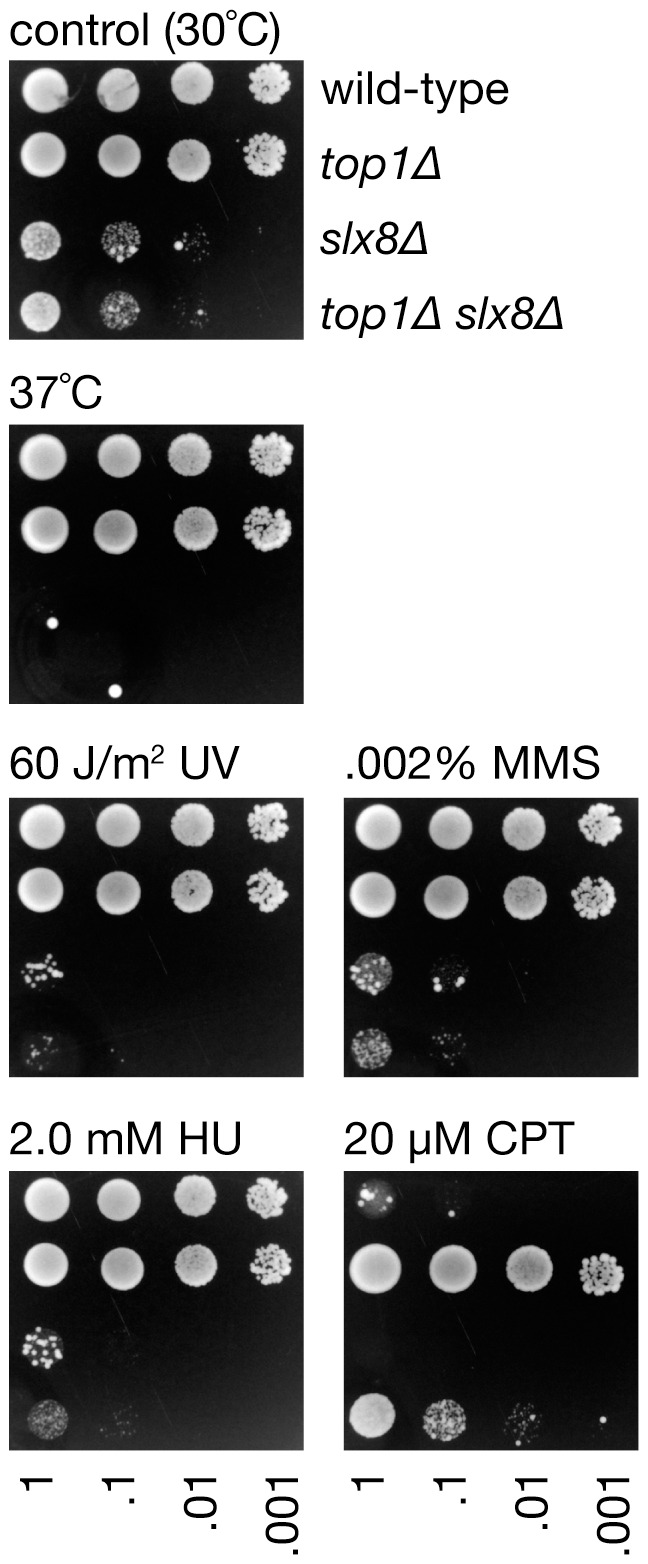
Spot assay comparing the growth and genotoxin sensitivity of strains MCW1221, MCW6516, FO986 and MCW6514. The plates were photographed after 5 days growth at 30°C unless otherwise indicated.

If the removal of Pli1 dependent Top1-SUMO conjugates by Slx8 promotes genome stability, then, similar to *pli1*Δ, the hyper-recombination of a *slx8*Δ mutant should be suppressed by deleting *top1*. Indeed, the deletion of *top1* does partially suppress (*P*<0.05) the increased *RTS1*-induced recombination in a *slx8*Δ mutant (Fig. 5B and Table S1), and this effect is not due to any reduction in *RTS1* barrier strength (Fig. 4C–D). In contrast, the spontaneous Ade^+^ recombinant frequency and ratio of deletion- to conversion-types is essentially the same in *slx8*Δ and *slx8Δ top1*Δ strains (Fig. 5A and Table S1). Moreover, apart from CPT hypersensitivity, deletion of *top1* does not suppress the poor growth and genotoxin hypersensitivity of a *slx8*Δ mutant (Fig. 7). These data indicate that removal of Top1-SUMO conjugates contributes to limiting local hyper-recombination at a programmed replication fork barrier but that accumulating Top1-SUMO conjugates are not the major cause of the spontaneous recombination and poor growth observed in Slx8 deficient cells.

## Discussion

We have shown that Pli1's role in limiting spontaneous direct repeat recombination is needed only in the presence of Top1, and is also negated in the vicinity of a blocked replication fork. Like Pli1, Slx8 is required for constraining direct repeat recombination, however this role is largely independent of Top1, and instead relates to a need for preventing the accumulation of Pli1-dependent SUMO conjugates.

### How does Pli1 limit spontaneous recombination?

How Pli1-dependent SUMOylation limits recombination in the presence of Top1 is unclear. One possibility is that SUMOylation of Top1 and/or other factors is needed for the removal of Top1cc, which could otherwise lead to replication fork stalling and breakage necessitating repair by HR involving both the Mus81-Eme1 structure-specific endonuclease and Rad51 [50], [51], [52]. Consistent with this model Pli1 deficient cells are dependent on Mus81 and Rad51 for viability in the presence of Top1 [11], [13], [23]. However, at present there is no direct evidence that Pli1 is involved in the removal of Top1cc, which in fission yeast appears to depend on either Tdp1 or a pathway involving Nse2, Rad60 and Slx8 [23]. In humans SUMOylation of Top1 is enhanced when it is trapped in the cleavage complex, and this might, along with other potential functions, play a role in helping to target its ubiquitination and subsequent degradation by the proteasome thereby enabling access for other DNA repair factors to repair the underlying single-strand break [23], [53], [54], [55], [56], [57]. A previous study found no evidence that Top1 is extensively SUMOylated in fission yeast [23], however we have shown that in a *slx8*Δ mutant a hyper-SUMOylated form of Top1 does accumulate and that this is dependent on Pli1. This suggests that Top1-SUMO conjugates are subject to removal by a STUbL-dependent mechanism. However, if this is the critical function for Pli1-dependent SUMOylation in limiting HR in the presence of Top1, then it seemingly does not depend on Top1 being conjugated to a SUMO chain as a *SUMO^K14/30R^* mutant, which is deficient in chain formation, is viable in the absence of Rad51 unlike a *pli1*Δ mutant [23]. Moreover, unlike Slx8, Nse2 and Rad60, Pli1 is not required for cell growth in the absence of Tdp1 and therefore cannot be an essential component of the alternative Top1cc removal pathway [23].

An alternative speculative idea for how Pli1 might prevent Top1-mediated HR derives from the recent finding that replication fork stalling at Top1cc results in fork regression [58]. In higher eukaryotes these regressed forks are stabilized by the poly(ADP-ribose) polymerase PARP-1, which interacts with the DNA helicase RECQ1 and in doing so prevents it from prematurely resetting the fork and thereby restarting replication [58], [59]. As there is no PARP in fission yeast it is conceivable that it uses Pli1-dependent SUMOylation as an alternative mechanism to manage replication fork stalling at Top1ccs so that restart occurs in a non-recombinogenic manner.

### Localized suppression of Pli1's antirecombinogenic role by *RTS1*

One of the intriguing results of our study is the localized suppression of *pli1*Δ hyper-recombination by replication fork blockage at *RTS1*. How this is achieved is unclear, but presumably involves either a localized reduction in the formation of Top1ccs or activation of an alternative pathway for limiting their recombinogenic impact. One way in which Top1cc formation could be reduced is if replication fork blockage at *RTS1* allows the dissipation of positive supercoils that build up ahead of the advancing fork. For example, cleavage of the stalled fork by Mus81-Eme1 would provide an alternative to Top1-mediated relaxation of supercoiled DNA [52]. However, in wild-type cells only a very small percentage of forks blocked at *RTS1* are subject to breakage [30], [33]; most are either resolved by passive replication from the opposing fork or restarted by a DSB-independent recombination process [31], [38], [40], [45]. Moreover, if there was a significant increase in fork breakage in a *pli1*Δ mutant we would expect to see an increase in recombinant formation and a greater proportion of deletion-types [22, unpublished data].

### Slx8's role in suppressing spontaneous recombination

Unlike Pli1, Slx8's main role in promoting genome stability is not to prevent unscheduled HR brought about by Top1 activity. This finding appears incongruous with the idea that Pli1's key role in preventing HR might be to target Top1 for Slx8-dependent degradation. However, it is conceivable that an overall increase in Pli1-dependent SUMO-conjugates in a *slx8*Δ mutant overrides any specific effect that it might have in failing to process SUMOylated Top1. Presumably either the persistence of specific SUMO conjugates and/or the decline in free SUMO (and consequent failure to SUMOylate proteins) results in the deregulation of HR or accumulation of DNA lesions that trigger it. Interestingly some of the hyper-recombination in a *slx8*Δ mutant at the *RTS1* RFB is suppressed in a Top1 mutant suggesting that in the vicinity of a blocked replication fork Slx8 may be needed for the removal of SUMOylated Top1 to avoid unnecessary HR.

### A note about SUMO chains

The function of SUMO chains remains largely enigmatic, and it is even unknown whether the majority of high molecular weight SUMO conjugates detected in yeast and higher eukaryotes represent chains that are attached or unattached to substrate proteins [60]. Our observation that two known SUMO targets (Top1 and tubulin) are part of the high molecular weight mass of SUMO conjugates that accumulate in a *slx8*Δ mutant suggests that SUMO chains may generally be attached to substrate proteins. However, whether this reflects a deliberate mechanism to regulate protein turnover in all cases, or simply a need to counter the effects of an overactive SUMOylation system is unclear [60].

### Is SUMOylation needed for proficient replication restart at *RTS1*?

There are a number of documented examples of how SUMOylation plays a role in both promoting and controlling HR. For example, in budding yeast Ubc9- and Mms21-dependent SUMOylation influences the ability of Sgs1 and Top3 to prevent the accumulation of recombinogenic structures on replicating chromosomes [61], and in human cells PIAS1- and PIAS4-dependent SUMOylation appears to target RNF4 to promote the stepwise progression of DSB repair by mediating the turnover of RPA bound to single-stranded DNA so that BRCA2 and RAD51 can take its place [62]. However, SUMOylation does not appear to be critical for HR-mediated replication restart in fission yeast. This assertion is based on our observation that neither *pli1*Δ nor *nse2-SA* mutant exhibit a significant reduction in the frequency of *RTS1*-induced recombination, which provides a readout for attempted replication restart of persistently stalled forks. It is also surprising that the deletion of both known E3 SUMO ligases does not manifest a more dramatic hyper-recombination phenotype, given that there are several proteins, which are likely to be influenced either directly or indirectly by SUMOylation, that strongly suppress *RTS1*-induced recombinant formation [45]. One example is the DNA helicase Srs2, which in budding yeast is recruited to stalled replication forks by SUMOylated PCNA and there acts to limit recombination by displacement of Rad51 and/or Polδ and Polη [63], [64], [65]. It should be noted that the majority of recombination induced by *RTS1* occurs accurately between sister chromatids and is therefore “genetically silent”, whereas only a minority occurs between the two *ade6^−^* heteroalleles and can therefore give rise to a genetically detectable recombinant. It is therefore possible that SUMOylation or a STUbL-dependent process is required for promoting both replication restart proficiency and fidelity, and the net effect of losing both these activities could be a recombinant frequency that is similar to wild-type. Clarification of this awaits further studies.

## Supporting Information

Table S1 **Direct repeat recombinant frequencies.**
(DOC)
