# Supplementary material for: Slx8 Removes Pli1-Dependent Protein-SUMO Conjugates Including SUMOylated Topoisomerase I to Promote Genome Stability
Source: PLoS One. 2013 Aug 6;8(8):e71960. doi: 10.1371/journal.pone.0071960 (PMC3735562; doi:10.1371/journal.pone.0071960)
Supplement: Table S1 — Direct repeat recombinant frequencies. (DOC) [file pone.0071960.s001.doc]

**Table S1:** Direct repeat recombinant frequencies

| Genotype | *RTS1* orientation | Number of colonies analysed^a^ | Total frequency of Ade+ recombinants (x 10^-4^)^b^ | Frequency of deletion-types  (x 10^-4^)^b^ | Frequency of conversion-types  (x 10^-4^)^b^ | Mean % conversion-types^b^ |
| --- | --- | --- | --- | --- | --- | --- |
| wild-type | 1 | 39 | 4.25  (+/- 0.88) | 2.97  (+/- 0.66) | 1.28  (+/- 0.39) | 30.02  (+/- 6.18) |
| *pli1∆* | 1 | 32 | 46.64  (+/- 13.92) | 33.58  (+/- 10.39) | 13.06  (+/- 5.11) | 28.17  (+/- 6.71) |
| *nse2-SA* | 1 | 15 | 6.41  (+/- 0.99) | 4.65  (+/- 0.86) | 1.76  (+/- 0.63) | 27.44  (+/- 7.77) |
| *pli1∆*  *nse2-SA* | 1 | 15 | 31.75  (+/- 11.85) | 22.36  (+/- 10.34) | 9.39  (+/- 3.71) | 30.6  (+/- 10.07) |
| *slx8∆* | 1 | 27 | 18.05  (+/- 5.36) | 15.31  (+/- 5.10) | 2.74  (+/- 1.09) | 15.70  (+/- 5.85) |
| *pli1∆ slx8∆* | 1 | 29 | 40.27  (+/- 10.78) | 27.88  (+/- 9.17) | 12.39  (+/- 3.37) | 31.54  (+/- 6.96) |
| *top1∆* | 1 | 18 | 3.55  (+/- 1.22) | 2.40  (+/- 0.90) | 1.15  (+/- 0.43) | 32.80  (+/- 6.37) |
| *pli1∆ top1∆* | 1 | 16 | 4.02  (+/- 1.63) | 3.08  (+/- 1.61) | 0.94  (+/- 0.47) | 24.70  (+/- 11.42) |
| *slx8∆ top1∆* | 1 | 27 | 19.60  (+/- 7.72) | 16.58  (+/- 6.41) | 3.02  (+/- 1.60) | 15.20  (+/- 3.53) |
| wild-type | 2 | 68 | 275.63  (+/- 71.79) | 116.08  (+/- 40.82) | 159.55  (+/- 40.28) | 58.41  (+/- 6.32) |
| *pli1∆* | 2 | 42 | 233.39  (+/- 41.29) | 112.49  (+/- 29.94) | 120.90  (+/- 24.14) | 52.13  (+/- 7.64) |
| *nse2-SA* | 2 | 22 | 279.35  (+/- 68.72) | 138.71  (+/- 38.82) | 140.64  (+/- 34.76) | 50.57  (+/- 4.6) |
| *pli1∆*  *nse2-SA* | 2 | 26 | 339.69  (+/- 113.06) | 183.61  (+/- 66.66) | 156.08  (+/- 56.19) | 46.11  (+/- 5.79) |
| *slx8∆* | 2 | 50 | 577.43  (+/- 135.50) | 355.01  (+/- 134.51) | 222.42  (+/- 58.79) | 39.72  (+/- 9.89) |
| *pli1∆ slx8∆* | 2 | 16 | 241.71  (+/- 59.54) | 109.02  (+/- 25.53) | 132.69  (+/- 38.48) | 54.60  (+/- 5.03) |
| *top1∆* | 2 | 18 | 220.00  (+/- 35.97) | 96.87  (+/- 25.09) | 123.13  (+/- 22.63) | 56.20  (+/- 7.56) |
| *pli1∆ top1∆* | 2 | 20 | 261.61  (+/- 127.29) | 152.92  (+/- 109.94) | 108.69  (+/- 38.39) | 48.29  (+/- 14.61) |
| *slx8∆ top1∆* | 2 | 21 | 422.32  (+/- 139.87) | 250.58  (+/- 120.14) | 171.74  (+/- 47.06) | 42.04  (+/- 9.28) |

^a^ At least 15 colonies from at least three independent experiments were analysed to derive the mean recombinant frequencies shown in this table, and for each colony at least 100 Ade+ recombinants were assessed for His+ prototrophy.

^b^ The values in parentheses are the standard deviations about the mean.
